# Interleukin-10 attenuation of collagen-induced arthritis is associated with suppression of interleukin-17 and retinoid-related orphan receptor γt production in macrophages and repression of classically activated macrophages

**DOI:** 10.1186/ar4544

**Published:** 2014-04-16

**Authors:** Liang Ye, Zhongyang Wen, Yanqun Li, Bingni Chen, Ting Yu, Lanying Liu, Jinshun Zhang, Yanmei Ma, Shuying Xiao, Liping Ding, Li Li, Zhong Huang

**Affiliations:** 1 Institute of Biological Therapy, Shenzhen University School of Medicine, Nanhai Ave 3688, Shenzhen, Guangdong 518060, China; 2 Department of Pathogen Biology and Immunology, Shenzhen University School of Medicine, Nanhai Ave 3688, Shenzhen, Guangdong 518060, China; 3 Shenzhen City Shenzhen University Immunodiagnostic Technology Platform, Nanhai Ave 3688, Shenzhen, Guangdong 518060, China

## Abstract

**Introduction:**

Our objective in the present study was to determine the signaling pathway of interleukin 10 (IL-10) for modulating IL-17 expression in macrophages and the importance of this mediation in collagen-induced arthritis (CIA).

**Methods:**

IL-10-knockout (IL-10^−/−^) mice and wild-type (WT) mice were immunized with chicken type II collagen (CII) to induce arthritis. The expression levels of IL-17 and retinoid-related orphan receptor γt (RORγt) in macrophages and joint tissues of IL-10^−/−^ and WT mice were analyzed by enzyme-linked immunosorbent assay, quantitative RT-PCR (qRT-PCR) and Western blotting. The F4/80 macrophages and positive IL-17-producing macrophages in synovial tissues of the mice were determined by immunohistochemistry. The populations of classically activated macrophage (M1) and alternatively activated macrophage (M2) phenotypes were analyzed by flow cytometry. The expression of genes associated with M1 and M2 markers was analyzed by qRT-PCR.

**Results:**

Compared to WT mice, IL-10^−/−^ mice had exacerbated CIA development, which was associated with increased production of T helper 17 cell (Th17)/Th1 proinflammatory cytokines and CII-specific immunoglobulin G2a antibody after CII immunization. Macrophages in IL-10^−/−^ mice had increased amounts of IL-17 and RORγt compared with the amounts in WT mice with CIA. Immunofluorescence microscopy showed that the number of IL-17-producing macrophages in synovial tissues was significantly higher in IL-10^−/−^ mice than in WT mice. IL-10 deficiency might promote macrophage polarization toward the proinflammatory M1 phenotype, which contributes to the rheumatoid arthritis inflammation response.

**Conclusion:**

IL-10 inhibits IL-17 and RORγt expression in macrophages and suppresses macrophages toward the proinflammatory M1 phenotype, which is important for the role of IL-10 in mediating the pathogenesis of CIA.

## Introduction

Rheumatoid arthritis (RA) is an inflammatory autoimmune disease characterized by chronic inflammation within the synovial tissues in multiple joints, and it leads to progressive, erosive destruction of cartilage and joints [1]. Collagen-induced arthritis (CIA) is a well-established animal model that has been studied extensively because of its similarities to human RA. Although the etiology and pathogenesis of RA have not been completely elucidated, an imbalance between pro- and anti-inflammatory cytokines has been reported to be a key mechanism for joint inflammation and disease progression in CIA as well as in human RA [2].

Interleukin 10 (IL-10) is an important immunoregulatory cytokine produced by many cell populations, including macrophages, dendritic cells (DCs), T-cell subsets (Th2, Tc2 and Tr1) and B cells [3]. Lipopolysaccharides (LPSs) induce the expression of IL-10 in macrophages *in vitro* and *in vivo*[4-6]. Many of the immunosuppressive characteristics of IL-10 can be traced to their effects on macrophages and DCs by preventing the production of the T helper 1 cell (Th1)–associated cytokines IL-2 and interferon γ (IFN-γ). The other profound effects of IL-10 are inhibition of the production of proinflammatory cytokines (IL-1, IL-6 and IL-12), inflammatory chemokines and matrix metalloproteases in macrophages [7]. IL-10 has been implicated to play a critical immunosuppressive role in autoimmune diseases, including RA [8-11]. In human RA, IL-10 suppresses the expression of tumor necrosis factor α (TNF-α), IL-1β and major histocompatibility complex (MHC) class II in macrophages in the synovial fluid of RA patients [12]. IL-10 also induces its own expression by human monocyte–derived macrophages. Thus, the anti-inflammatory response mediated by IL-10 provides pivotal regulation of suppression of autoimmune disease development.

Several immune cell types play a role in the pathogenesis of RA, such as fibroblasts, T lymphocytes, B lymphocytes and macrophages [13]. Macrophages appear to play a critical role in RA development because they are numerous in the inflamed synovial membrane and at the cartilage–pannus junction [14]. They also possess broad proinflammatory and destructive potential by expressing amounts of the inflammatory cytokines IL-1, TNF-α and IL-6 and by producing matrix metalloproteinases [15]. Activated macrophages can be broadly classified into classically activated macrophages (M1) and alternatively activated macrophages (M2). Typical stimuli for the M1 macrophages are IFN-γ and LPS, and typical stimuli for the M2 phenotype are IL-4 and IL-13 [16]. In general, M1 macrophages express high levels of TNF-α, IL-1β, IL-6, IL-12, IL-23 and type I IFN, as well as expressing inducible nitric oxide synthase (iNOS), chemokine (C-X-C motif) ligand 9 (CXCL9), CXCL10, CXCL11, C-C chemokine receptor type 7 (CCR7) and human leukocyte antigen, MHC class II molecule, DR isotype. M2 macrophages express high levels of IL-13, IL-1 receptor antagonist (IL-1ra), IL-4 and IL-10, as well as expressing mannose receptor CD206, chitinase 3–like 3, cluster of differentiation 163 (CD163), resistin-like molecule α1, arginase 1 and chitotriosidase [17-19]. Thus, M1 and M2 macrophages promote Th1 and Th2 responses, respectively [16]. Recently, a key role for macrophages in RA development has been suggested in part by successful treatment of RA by anti-TNF antibodies, because TNF is widely considered to be produced by activated macrophages in inflammatory tissues [20]. Although the anti-inflammatory effects of IL-10 in CIA have been shown in T cells, but those in macrophages have not yet been studied. Furthermore, the precise IL-10 signaling pathway in macrophages for the inhibition of the pathogenesis of CIA remains to be illuminated.

Recently, IL-17 (also known as IL-17A) is considered the signature cytokine in the Th17 cell population and has been implicated as having a role in the pathogenesis of numerous autoimmune diseases, including RA [21]. In RA, high levels of IL-17 and its receptor are found in RA synovial fluid and tissues [21]. IL-17-transgenic mice are prone to develop CIA [22], whereas inhibition of IL-17 and its receptors with antibodies delay the development of arthritis and reduce its consequences [23]. Researchers in previous studies have suggested that the IL-23/IL-17 pathway, rather than the IL-12–IFN-γ axis, is essential to promoting the development of CIA [24]. It has been reported that IL-10 suppresses IL-17 expression by CD11b^+^ cells and T cells in *in vitro* culture [25]. However, whether IL-10 regulates IL-17 expression in macrophages from CIA *in vivo* has not been studied.

In our present study, we investigated the functions of IL-10 in RA. IL-10-knockout (IL-10^−/−^) mice and their WT counterparts were used to establish a RA model. The results show that the development of CIA is exacerbated in IL-10^−/−^ mice. Macrophages in IL-10^−/−^ mice significantly upregulate the expression of IL-17 and retinoid-related orphan receptor γt (RORγt) *in vivo* and *in vitro*. Moreover, IL-10^−/−^ macrophages might enhance the M1 macrophage–mediated proinflammatory response, which accelerates the RA inflammation response.

## Methods

### Mice

Male C57BL/6 mice, wild-type (WT) mice and C57BL/6 IL-10^−/−^ mice were purchased from The Jackson Laboratory (Bar Harbor, ME, USA). The mice were housed under specific pathogen-free conditions. All animal experiments were approved by the Institutional Animal Care and Use Committee of Shenzhen University. We used mice at 10 to 14 weeks of age for experiments.

### Collagen-induced arthritis induction

CIA induction was performed according to a previously described protocol with minor modifications [26,27]. Briefly, male IL-10^−/−^ mice and WT mice were immunized intradermally at the base of the tail with 200 μl of chicken type II collagen (CII) (Chondrex, Redmond, WA, USA) emulsified in complete Freund’s adjuvant containing *Mycobacterium tuberculosis* (Chondrex). On day 14, these mice were given a second injection of CII dissolved in complete Freund’s adjuvant. Clinical arthritis was evaluated using the following scale: grade 0 = no swelling; grade 1 = slight swelling and erythema; grade 2 = pronounced swelling; and grade 3 = joint rigidity.

### Preparation of peritoneal macrophages

The mice were injected intraperitoneally with 2 ml of 5% thioglycollate medium (Sigma-Aldrich, St Louis, MO, USA) for 3 days. They were then killed, and peritoneal macrophages were isolated by lavage with phosphate-buffered saline (PBS). The cells were cultured in Dulbecco’s modified Eagle’s medium containing 10% fetal bovine serum (FBS) and antibiotics for 2 hours. Next, the cell cultures were washed to remove nonadherent cells before stimulation, and an aliquot was stained with F4/80 and sorted by flow cytometry.

### Preparation of joint macrophages and collection of synovial fluid

Joint macrophages and synovial fluid were collected according to a previously described protocol [28,29]. Briefly, after excision of the skin and patellar ligament under a dissecting microscope to expose the synovial membrane, a 30-gauge needle (BD Biosciences, San Jose, CA, USA) was carefully inserted into the membrane, and the synovial cavity was washed by repetitive injections and aspirations with PBS (20 μl) to obtain synovial lavage material. This procedure was repeated five times, and a total volume of 100 μl of synovial lavage fluid was obtained. After that step, joint and paws samples were removed and kept in RPMI 1640 medium (HyClone Laboratories/Thermo Fisher Scientific, Logan, UT, USA) containing 10% FBS (Sijiqing, Zhejiang, China), 100 IU/ml penicillin, 100 μg/ml streptomycin (Beyotime Institute of Biotechnology, Shanghai, China) and 1 mg/ml collagenase (Sigma-Aldrich). The entire mixture was minced and incubated for 1 hour at 37°C in a 5% CO_2_ atmosphere. The procedure was repeated three times, and cell suspensions were filtered with a cell strainer after red blood cell lysis. For macrophage isolation, the total of the above-described cell suspensions in a six-well plate for 2 hours and the adherent cells were harvested as joint macrophages. Synovial fluid samples were stored at −80°C prior to performing assays.

### Quantitative RT-PCR analysis

RNA samples were extracted by using TRIzol reagent (Invitrogen, Carlsbad, CA, USA), and cDNA was prepared by using the iScript cDNA Synthesis Kit (Bio-Rad Laboratories, Hercules, CA, USA). Quantitative RT-PCR (qRT-PCR) was performed to measure IL-17, RORγt, TNF-α, IL-1β, IL-6, iNOS, IL-13, CD206 and IL-1ra, respectively. The PCR primers used for qRT-PCR were as follows: IL-17, 5′-CAGCAGCGATCATCCCTCAAAG-3′ (forward) and 5′-CAGGACCAGGATCTCTTGCTG-3′ (reverse); for RORγt, 5′-CCGCTGAGAGGGCTTCA-3′ (forward) and 5′-TGCAGGAGTAGGCCACATTACA-3′ (reverse); for TNF-α, 5′-CAAAGGGAGAGTGGTCAGGT-3′ (forward) and 5′-GGCAACAAGGTAGAGAGGC-3′ (reverse); for IL-1β, 5′-CCTTCCAGGATGAGGACATGA-3′ (forward) and 5′-TGAGTCACAGAGGATGGGCTC-3′ (reverse); for IL-6, 5′-ATGGATGCTACCAAACTGGAT-3′ (forward) and 5′-TGAAGGACTCTGGCTTTGTCT-3′ (reverse); for iNOS, 5′-ACATCGACCCGTCCACAGTAT-3′ (forward) and 5′-CAGAGGGGTAGGCTTGTCTC-3′ (reverse); for IL-13, 5′-TGAGCAACATCACACAAGACC-3′ (forward) and 5′-GGCCTTGCGGTTACAGAGG-3′ (reverse); for CD206, 5′-GGCAGGATCTTGGCAACCTAGTA-3′ (forward) and 5′-CCTTTC TTCCGACTCTTCACCC-3′ (reverse); for IL-1ra, 5′-ACAGTAGAAGGAGACAGAAG-3′ (forward) and 5′-GGTGGTAGAGCAGAAGAC-3′ (reverse); and for β-actin, 5′-GTGACGTTGACATCCGTAAAGA-3′ (forward) and 5′-GCCGGACTCATCGTACTCC-3′ (reverse). IL-17, RORγt, TNF-α, IL-1β, IL-6, iNOS, IL-13, CD206 and IL-1ra transcript levels were measured using an *Applied Biosystems 7500* Fast Real-Time PCR *System* with SYBR Green PCR Master Mix according to the instructions of the manufacturer (Applied Biosystems, Foster City, CA, USA) and as described previously [30]. Relative expression levels of target genes were calculated with normalization to β-actin values using the 2^−ΔΔCt^ comparative cycle threshold method.

### Confocal and immunofluorescence microscopy

Peritoneal and joint macrophages were fixed with 4% paraformaldehyde for 10 minutes. After being washed with PBS three times for 5 minutes each, the specimens were permeabilized with 0.1% Triton X-100 in PBS for 5 minutes. Next, they were again washed three times for 5 minutes each with PBS. Nonspecific binding of antibodies was blocked by incubating the coverslips with 3% bovine serum albumin (BSA) for 12 hours. After being washed three times with PBS for 5 minutes each, the cells were then incubated with anti-F4/80 fluorescein isothiocyanate (FITC) antibody at 1:100 (eBioscience, San Diego, CA, USA), Alexa Fluor 647 IL-17 antibody at 1:100 (BD Biosciences) and RORγt phycoerythrin (PE) antibody at 1:100 (BD Biosciences) in 3% BSA at 37°C for 1 hour. After being washed another three times with PBS, nuclei were counterstained with 4′,6-diamidino-2-phenylindole (DAPI) at 1:1,000 for 5 minutes. Peritoneal macrophage samples were examined under an Olympus fluorescence microscope (Olympus America, Center Valley, PA, USA). Joint macrophages samples were analyzed under a Leica TCS SP5 confocal laser-scanning fluorescence microscope (Leica Microsystems, Buffalo Grove, IL, USA).

### Enzyme-linked immunosorbent assay

Serum and synovial fluid samples were collected from different groups of immunized and nonimmunized mice on day 45 after CII immunization. The levels of IL-17, IFN-γ, IL-6 and IL-1β were measured by enzyme-linked immunosorbent assay (ELISA) using an eBioscience kit. CII-specific immunoglobulin G1 (IgG1) and IgG2a antibodies (eBioscience) were detected by ELISA as previously described [9].

### Western blot analysis

Macrophages and total joint tissue proteins were extracted from CIA mice. For Western blotting, we used the following antibodies: anti-IL-17 (Abcam, Cambridge, MA, USA), anti-RORγt (eBioscience) and anti-β actin (Cell Signaling Technology, Danvers, MA, USA). Immune reaction bands were detected by using horseradish peroxidase–labeled, species-specific secondary antibodies (Cell Signaling Technology) and enhanced chemiluminescence analysis (EMD Millipore, Billerica, MA, USA). Immune reaction bands were scanned using Kodak Image Station 4000MM (Eastman Kodak, Rochester, NY, USA), and images were quantified using Quantity One 1-D Analysis software (Bio-Rad Laboratories).

### Flow cytometric analysis

Joint macrophages were prepared and filtered with a cell strainer. F4/80 macrophages were used as pan-macrophage markers, iNOS was used as a marker of M1 macrophages and CD206 was used as a marker of M2 macrophages [18,31,32]. Surface staining was performed using the following monoclonal antibodies: anti-F4/80 FITC (eBioscience), anti-CD206 PE (BioLegend, San Diego, CA, USA). Anti-iNOS allophycocyanin was purchased from BD Biosciences. For intracellular staining of iNOS, Cell Stimulation Cocktail (a cocktail of phorbol 12-myristate 13-acetate, ionomycin, brefeldin A and monensin from eBioscience) was added and cultured for the last 5 hours before flow cytometric analysis as previously described [25].

### Immunofluorescence histochemistry

IL-17^+^ and F4/80^+^ macrophages in the knee joints were analyzed by immunofluorescence staining of frozen joint sections from IL-10^−/−^ and WT mice with or without CII immunization. Briefly, knee joint samples were fixed with 4% paraformaldehyde, decalcified with 14% ethylenediaminetetraacetic acid and embedded in optimal cutting temperature compound with 30% sucrose. Macrophages were stained with fluorochrome-conjugated anti-F4/80 FITC antibody (eBioscience) and Alexa Fluor 647 IL-17 antibody (BD Biosciences) in a dark chamber for 30 minutes at room temperature. Nuclei were counterstained with DAPI (1 μg/ml) for 5 minutes. Slides were examined using an Olympus fluorescence microscope.

### Histopathological analysis

Paraffin-embedded knee joint tissue sections (5 μm thick) were stained with hematoxylin and eosin. Histopathologic scoring of joint damage was performed under blinded conditions according to a scoring system widely used for evaluating synovitis, cartilage degradation and bone erosion [33].

### Statistical analysis

We used GraphPad version 5.0 software (GraphPad Software, La Jolla, CA, USA) to calculate the mean and SEM values for statistical tests. Comparisons between the WT and IL-10^−/−^ groups were analyzed by performing a two-tailed, unpaired Student’s *t*-test. *P*-values <0.05 were considered statistically significant.

## Results

### IL-10^−/−^ exacerbates collagen-induced arthritis development in mice

**Figure 1:**
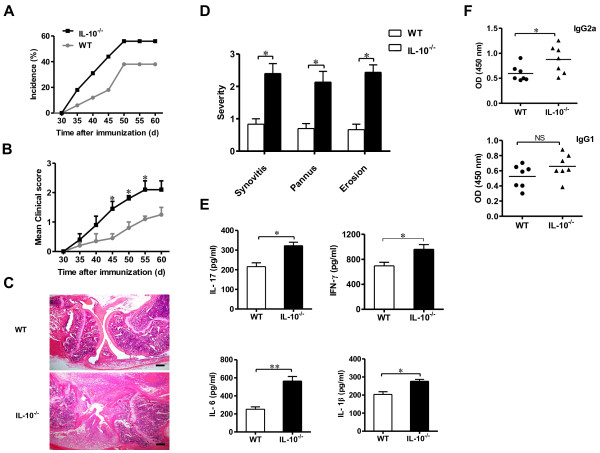
**Interleukin 10–knockout mice were highly susceptible to collagen-induced arthritis. (A)** Graph illustrating the incidence of arthritis in interleukin 10–knockout (IL-10^−/−^) and wild-type (WT) mice. The incidence values were recorded on day (d) 30 after chicken type II collagen (CII) immunization. Values are representative of five independent experiments (*n* = 9 per group in each experiment). The mice were killed on day 45 or later postimmunization for immunological analysis and histopathologic examination. **(B)** Graphed mean paw clinical arthritis severity scores (maximum possible score = 3) in IL-10^−/−^ mice and WT mice with collagen-induced arthritis (CIA). Values are mean ± SEM (*n* = 45). *P < 0.05 vs. WT mice. **(C)** Histopathology images of the knee joints of IL-10^−/−^ mice and WT mice with CIA (hematoxylin and eosin stain; original bar length = 200 μm). **(D)** Graphed evaluation results for synovitis, pannus and erosion of bone and cartilage in knee joint sections taken from IL-10^−/−^ and WT arthritic mice (*n* = 3). **(E)** On day 45 following immunization, IL-10^−/−^ and WT arthritic mice were bled and the concentrations of serum cytokines (IL-17, interferon γ (IFN-γ), IL-6 and IL-1β) of individual mice were determined by enzyme-linked immunosorbent assay (ELISA) (*n* = 3). **(F)** On day 45 following immunization, IL-10^−/−^ and WT arthritic mice were bled and the serum levels of CII-specific immunoglobulin G2a (IgG2a) and IgG1 antibodies were measured by ELISA (*n* = 7). OD = Optical density. Horizontal lines indicate mean values. Data in (D) through (F) are mean ± SEM. *P < 0.05; **P < 0.01. NS = Not significant.

To investigate the role of IL-10 in the pathogenesis of C57BL/6 mice with CIA, male IL-10^−/−^ and WT mice were immunized with CII to induce arthritis. On day 45 postimmunization, RA incidence and paw clinical scores were markedly higher in IL-10^−/−^ mice than in WT mice (Figures 1A and B). Furthermore, IL-10^−/−^ mice had more serious synovial hyperplasia, cartilage damage and bone erosion than WT mice in the histopathologic examination of joints (Figure 1C and D).

To investigate the anti-inflammatory effect of IL-10 on CIA, proinflammatory cytokines in serum were detected by ELISA. The amounts of IL-17, IFN-γ, IL-6 and IL-1β in IL-10^−/−^ mice were markedly higher than those in WT mice (Figure 1E). To verify whether the reason why IL-10^−/−^ mice had more severe CIA was due to modulation of the humoral immune response against CII, serum from immunized WT and IL-10^−/−^ mice were analyzed for the presence of CII-specific IgG1 and IgG2a antibody isotypes. Compared to WT mice, IL-10^−/−^ mice had significantly higher serum levels of anti-CII IgG2a antibodies (Figure 1F), but we found no significant differences in the levels of IgG1 in the serum of WT mice and IL-10^−/−^ mice (Figure 1F). The higher proinflammatory cytokines and specific CII IgG2a antibody secretion, as well as the greater susceptibility and severity of disease, in IL-10^−/−^ mice suggests that IL-10 probably plays a central role in the regulation of CIA development.

### Increased joint synovial fluid and tissue IL-17 and RORγt expression in IL-10^−/−^ mice with CIA

**Figure 2:**
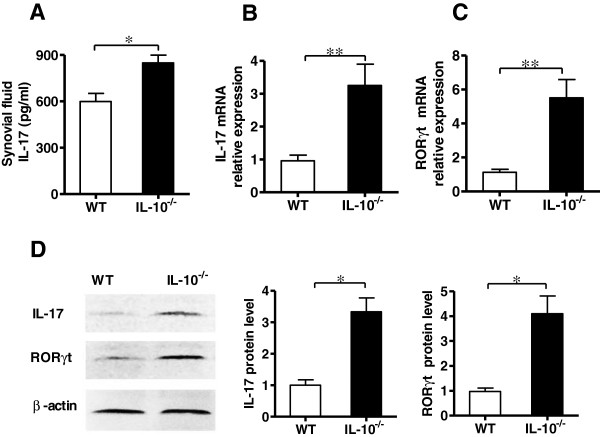
**Interleukin 10 suppresses the expression of interleukin 17 and retinoid-related orphan receptor γt in local joints of mice with collagen-induced arthritis. (A)** The levels of interleukin 17 (IL-17) in the knee joint synovial fluid of IL-10-knockout (IL-10^−/−^) mice and wild-type (WT) mice with collagen-induced arthritis (CIA) were measured by using an enzyme-linked immunosorbent assay (*n* = 5). **(B)** Expression levels of IL-17 mRNA in synovial tissue of IL-10^−/−^ mice and WT mice with CIA were measured by performing quantitative RT-PCR (qRT-PCR) experiments (*n* = 5). **(C)** Expression levels of retinoid-related orphan receptor γt (RORγt) mRNA in synovial tissues of IL-10^−/−^ mice and WT mice with CIA were measured by qRT-PCR (*n* = 5). **(D)** The protein levels of IL-17 and RORγt in synovial tissues of IL-10^−/−^ mice and WT mice with CIA were determined by Western blot analysis and then quantified by densitometry. β-actin was used as an internal control (*n* = 3). Data in **(A)** through **(D)** were obtained on day 45 after type II collagen immunization and are mean ± SEM. *P < 0.05; **P < 0.01.

The pathogenesis of arthritis is dependent upon the secretion of proinflammatory cytokines and the subsequent recruitment of inflammatory cells into synovial tissues [2]. IL-17 is a critical cytokine in the pathogenesis of CIA [21]. To determine whether IL-10 signaling involved in the regulation of Th17 immune response in local joints of CIA mice, the levels of IL-17 were measured in synovial fluid and tissues. The ELISA results showed that IL-10^−/−^ mice had significantly higher amounts of IL-17 than WT mice (Figure 2A). qRT-PCR also demonstrated that the transcription of IL-17 was elevated (approximately 3.4-fold) in IL-10^−/−^ mice synovial tissue compared with IL-10^+/+^ mice (Figure 2B).

The orphan nuclear receptor RORγt is a key transcription factor that induces transcription of the genes encoding IL-17 [34]. It has been well-documented that IL-10 plays an important role in the inhibition of IL-17 expression, but whether the downregulation of IL-17 by IL-10 is through the inhibition of RORγt is not known. To study the relationships between IL-10 and RORγt, we examined the expression of RORγt in IL-10^−/−^ and WT mice with CIA. As predicted, IL-10^−/−^ mice had clearly (approximately 4.9-fold) higher RORγt mRNA expression than WT mice in synovial tissue (Figure 2C). Western blot analysis further demonstrated that joint synovial tissues of IL-10^−/−^ mice had much higher levels of IL-17 and RORγt proteins than those of WT mice (Figure 2D). Collectively, our results indicate that IL-10 suppresses IL-17 expression and RORγt transcription in synovial tissues of CIA mice.

### IL-17 expression was enhanced *in vitro* in F4/80^+^ macrophages of IL-10^−/−^ mice

**Figure 3:**
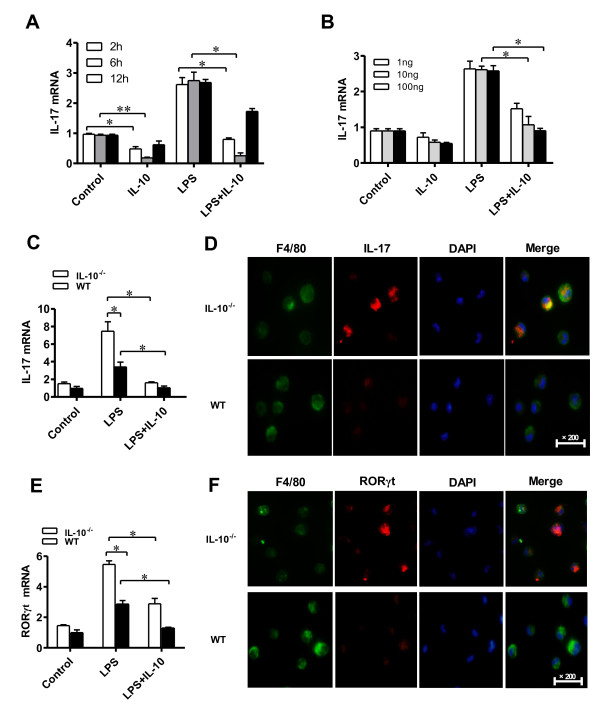
**Interleukin 10 inhibits the expression of interleukin 17 in macrophages in vitro*. *(A)** Thioglycollate-elicited peritoneal (TEP) macrophages from interleukin 10–knockout (IL-10^−/−^) mice were activated with or without 1 μg/ml lipopolysaccharide (LPS) in culture for 1 hour, followed by addition of 100 ng/ml IL-10, and then cultured for 2, 6 and 12 hours. Total RNA was extracted and analyzed for IL-17 mRNA by quantitative RT-PCR (qRT-PCR). **(B)** TEP macrophages from IL-10^−/−^ mice were activated with or without LPS (1 μg/ml) for 1 hour, and then 1, 10 or 100 ng/ml IL-10 was added separately to cultures. The cells were cultured for another 6 hours, and total RNA was extracted and analyzed for IL-17 mRNA by qRT-PCR. **(C)** and **(E)** TEP F4/80^+^ macrophages from IL-10^−/−^ mice and WT mice were separated by flow cytometry and then cultured and stimulated with LPS for 1 hour in the presence or absence of 100 ng/ml IL-10 for 6 hours. Total RNA was extracted and analyzed in qRT-PCR experiments for IL-17 mRNA **(C)** and retinoid-related orphan receptor γt (RORγt) mRNA **(E)**. **(D)** and **(F)** TEP macrophages from IL-10^−/−^ mice and WT mice were activated with 1 μg/ml LPS for 1 hour, F4/80^+^ macrophages were stained with fluorescein isothiocyanate-conjugated F4/80 (green) and AleaxFluor647-conjugated IL-17 (red) for IL-17 **(D)** and RORγt **(F)** expression and analyzed by immunofluorescence microscopy. Original magnification, ×200. Values are mean ± SEM (*n* = 3). *P < 0.05; **P < 0.01.

How IL-10 regulates IL-17 expression in macrophages remains to be resolved. First, we analyzed the effect of IL-10 on IL-17 expression at different time points. Thioglycollate-elicited peritoneal macrophages purified from WT mice were cultured with or without LPS (1 μg/ml) in cultures for 1 hour and then with or without IL-10 (100 ng/ml) for 2, 6 and 12 hours in each condition. Compared to LPS alone, when IL-10 was introduced into the cultures, IL-17 mRNA expression was significantly reduced at 2 and 6 hours, but almost recovered at 12 hours, and the peak inhibition for IL-17 expression by IL-10 occurred at 6 hours (Figure 3A). Second, we tested the effect of IL-10 on IL-17 expression at different concentrations. IL-10 at 1, 10 and 100 ng/ml concentrations was added to the cultures for 6 hours after 1-hour LPS stimulation. At concentrations of 10 ng/ml and 100 ng/ml IL-10, IL-17 mRNA expression was reduced 2.4- and 2.7-fold, respectively (Figure 3B). Overall, our results indicate that IL-10 restrains the expression of IL-17 in macrophages.

Considering the purity of macrophages in the group of CD11b^+^ cells, F4/80 macrophages (shown to be specific markers for macrophages) were isolated by flow cytometry from a thioglycollate-elicited peritoneal cell population in IL-10^−/−^ and WT mice. The resulting cells were used to study the regulation of IL-17 expression by IL-10. The experiments showed that after LPS stimulation for 1 hour, IL-10^−/−^ mice F4/80 macrophages had significantly higher IL-17 mRNA expression (2.1-fold) than WT mice F4/80^+^ macrophages with the same treatment used (Figure 3C). The inhibition of IL-17 in F4/80^+^ macrophages by IL-10 was confirmed by immunofluorescence microscopy (Figure 3D). To further address the effect of IL-10 on IL-17 expression in F4/80^+^ macrophages, IL-10 (100 ng/ml) was added to the cultures of WT and IL-10^−/−^ F4/80^+^ macrophages, respectively. The results of the experiments showed that the expression of IL-17 was nearly totally suppressed by IL-10 in both WT and IL-10^−/−^ F4/80^+^ macrophages (Figure 3C). Collectively, these results further confirm that the expression of IL-17 is inhibited by IL-10 in F4/80^+^ macrophages.

Of note, RORγt is the key transcription factor for IL-17. Because IL-10 inhibits IL-17 expression in F4/80^+^ macrophages, we next examined the expression of RORγt in the F4/80^+^ macrophages of IL-10^−/−^ mice. As predicted, the expression of RORγt was strongly induced by LPS in F4/80^+^ macrophages of both WT and IL-10^−/−^ mice, but its expression was more significant in F4/80^+^ macrophages from IL-10^−/−^ mice. Compared to WT, the expression of RORγt was strongly induced (1.9-fold) by LPS in F4/80^+^ macrophages in IL-10^−/−^ mice (Figure 3E). The enhanced RORγt expression by LPS in F4/80^+^ macrophages was inhibited by the addition of exogenous IL-10 (Figure 3E). Furthermore, the suppression of RORγt expression in F4/80^+^ macrophages was confirmed by immunofluorescence microscopy (Figure 3F). Taken together, these results indicate that IL-10 is a pivotal cytokine controlling IL-17 and RORγt expression in macrophages *in vitro.*

### IL-17 expression in joint synovium *in vivo* was increased in macrophages from IL-10^−/−^ mice with collagen-induced arthritis

**Figure 4:**
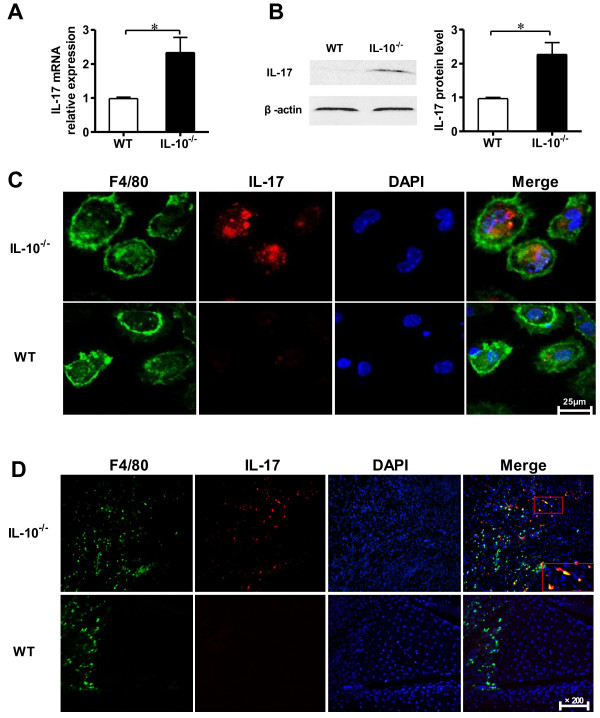
**Interleukin 10 inhibits interleukin 17 production by macrophages in mice with collagen-induced arthritis. (A)** Macrophages in the joint tissues and synovial fluid (SF) taken from interleukin 10–knockout (IL-10^−/−^) mice and wild-type (WT) mice with collagen-induced arthritis (CIA) on day 45 after chicken type II collagen (CII) immunization were isolated. The total cellular RNA was extracted and analyzed for IL-17 mRNA by quantitative RT-PCR. Data are mean ± SEM (*n* = 3). *P < 0.05. **(B)** Macrophage proteins were extracted, and Western analysis was performed to detect protein levels of IL-17 and then quantified by densitometry. β-actin was used as an internal control. Data are mean ± SEM (*n* = 3). *P < 0.05. **(C)** Macrophages in the joint tissues and SF from IL-10^−/−^ mice and WT mice with CIA on day 45 post–CII immunization were isolated, and cells were stained with fluorescein isothiocyanate–conjugated F4/80 (green) or Alexa Fluor 647–conjugated IL-17 (red) and analyzed by using confocal microscopy Bar = 25 μm. **(D)** In the joint synovium taken from IL-10^−/−^ mice and WT mice with CIA, macrophages were detected by fluorescein isothiocyanate–conjugated F4/80 (green), Alexa Fluor 647–conjugated IL-17 (red) or immunofluorescence double-staining of frozen sections (original magnification, ×200). Boxed areas highlight IL-17/F4/80 double-positive macrophages in the joint synovium.

It has been well-documented that IL-17 plays an important role in the development of CIA, but the effects of IL-10 on IL-17 expression in macrophages from CIA have not yet been elucidated. To study the relationship between IL-10 and IL-17 in macrophages in CIA, we examined the expression of IL-17 in joint synovial macrophages from IL-10^−/−^ and WT mice with CIA. The expression levels of IL-17 mRNA and protein were detected by qRT-PCR and Western blot analysis, respectively. Compared to WT mice, IL-10^−/−^ mice had significantly increased expression of IL-17 mRNA and protein (approximately 2.3-fold and about 2.4 fold, respectively) in macrophages of joint synovial tissues in CIA (Figures 4A and 4B). In addition, the expression of IL-17 in macrophages was confirmed by confocal immunofluorescence microscopy (Figure 4C). Further examination of frozen joint sections by immunofluorescence microscopy indicated that IL-10^−/−^ mice had substantially increased IL-17-producing macrophages in synovial tissue compared with WT mice (Figure 4D). These results demonstrate that IL-10 suppresses IL-17 expression in synovial macrophages in CIA, which implies that the inhibition of RA inflammation by IL-10 might occur through the regulation of IL-17 expression in the synovium.

### Mechanism of IL-10 inhibits joint synovium IL-17 expression in macrophages in collagen-induced arthritis

**Figure 5:**
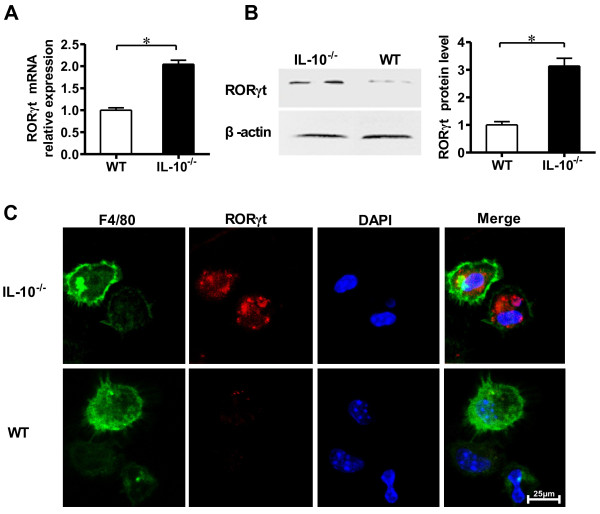
**Interleukin 10 suppresses retinoid-related orphan receptor γt expression in macrophages of mice with collagen-induced arthritis. (A)** Macrophages in the joint tissues and synovial fluid (SF) taken from interleukin 10–knockout (IL-10^−/−^) mice and wild-type (WT) mice with collagen-induced arthritis (CIA) on day 45 after chicken type II collagen (CII) immunization were then isolated. The total cellular RNA was extracted and analyzed for retinoid-related orphan receptor γt (RORγt) mRNA by quantitative RT-PCR. Data are mean ± SEM (*n* = 3). *P < 0.05. **(B)** Macrophage proteins were extracted and Western blot analysis was performed to detect protein levels of RORγt, which were then quantified by densitometry. β-actin was used as an internal control. Data are mean ± SEM (*n* = 3). *P < 0.05. **(C)** Macrophages isolated from the joint tissues and SF of IL-10^−/−^ and WT mice with CIA on day 45 after immunization were stained with fluorescein isothiocyanate–conjugated F4/80 (green) or phycoerythrin-conjugated RORγt (red) and analyzed by confocal microscopy. Bar = 25 μm.

After we obtained the results described above, we asked how IL-10 signaling mediates the expression of IL-17 in macrophages in CIA. To study this problem, we investigated whether IL-10 regulates transcription factor RORγt expression in joint synovium macrophages in CIA. qRT-PCR showed that expression of RORγt mRNA in macrophages was significantly higher (approximately 2.2-fold) in IL-10^−/−^ mice than in WT mice (Figure 5A). Furthermore, the level of RORγt transcription was much higher (about 3.2-fold) in IL-10^−/−^ mice macrophages than in WT mice macrophages (Figure 5B). In addition, the expression of RORγt in macrophages was further confirmed by confocal immunofluorescence microscopy (Figure 5C). These experiments indicated that restrained IL-17 expression by IL-10 in CIA joint macrophages is associated with suppression of the expression of RORγt.

### IL-10 suppresses macrophage polarization toward M1 phenotype and inhibits M1 phenotypic marker expression in CIA

**Figure 6:**
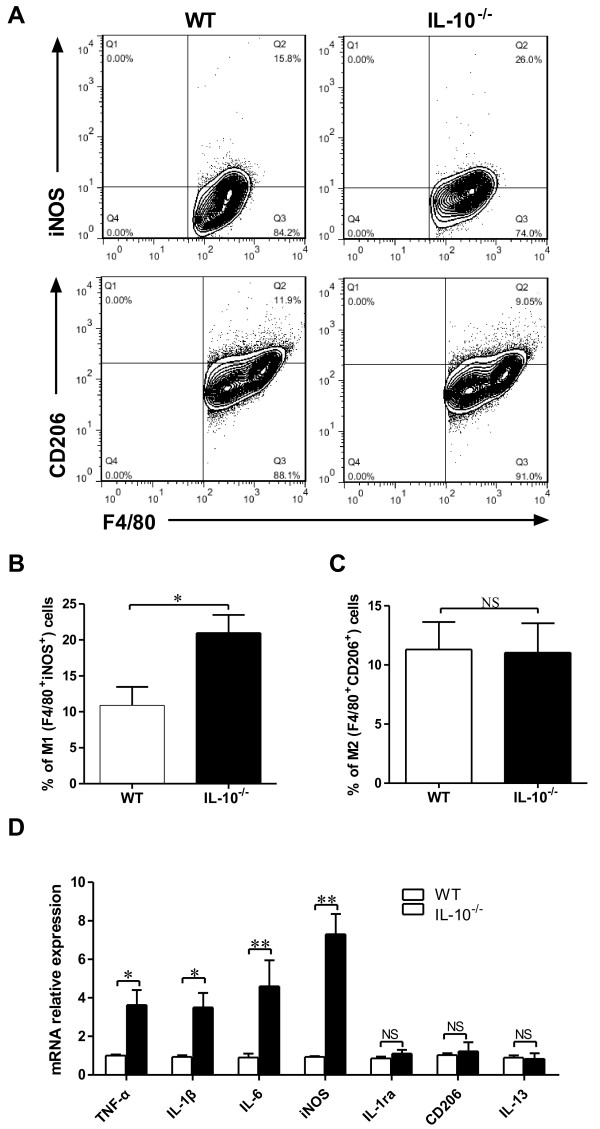
**Interleukin 10 polarizes macrophages toward the M1 phenotype and inhibits M1 marker expression in mice with collagen-induced arthritis.** Joint macrophages from interleukin 10–knockout (IL-10^−/−^) mice and wild-type (WT) mice with collagen-induced arthritis (CIA) on day 45 after chicken type II collagen (CII) immunization were isolated. Cells positive for macrophage marker F4/80 detected by flow cytometry were defined as macrophages. Within that population, macrophages were analyzed using inducible nitric oxide synthase (iNOS) as a marker of the classically activated macrophage (M1) phenotype and CD206 as a marker of the alternatively activated macrophage (M2) phenotype. **(A)** Percentage of M1 macrophages (F4/80^+^iNOS^+^) and M2 macrophages (F4/80^+^CD206^+^) of IL-10^−/−^ mice and WT mice with CIA assessed by flow cytometry. **(B)** and **(C)** Total M1 and M2 cells were quantified. **(D)** The total cellular RNA was extracted and analyzed for the expression of genes associated with M1 and M2 macrophage markers by quantitative RT-PCR. Data are mean ± SEM (*n* = 3). *P < 0.05; **P < 0.01. NS = Not significant.

IL-10 has also been reported to promote the differentiation of M2 macrophages and block the differentiation of M1 macrophages *in vitro*[19,35]. Wu *et al*. confirmed that exogenous IL-10 represses the M1 macrophage phenotype and suppresses M1-mediated proinflammatory cytokine production [36]. The data produced in our present study indicate that the expression of M1-associated cytokines IFN-γ, IL-6 and IL-1β is also increased in IL-10^*−/−*^ mice with CIA (Figure 1). However, it was unclear whether IL-10^*−/−*^ macrophages would express predominantly M1 or M2 profiles in CIA. To address this issue, we analyzed the joint macrophage phenotype in the IL-10^*−/−*^ and WT mice with CIA. *Macrophages* were defined as cells positive for the macrophage marker F4/80. Within the macrophage population, M1 macrophages were defined as iNOS-positive cells and M2 macrophages were defined as CD206-positive cells [18,31,32]. Compared to WT mice, both the percentage and the total number of M1 (F4/80^+^ and iNOS^+^) cells were significantly expanded in IL-10^*−/−*^ mice with CIA (Figures 6A and 6B), but the percentage and the total number of M2 (F4/80^+^ and CD206^+^) cells were not altered (Figures 6A and 6C). Next, we determined the mRNA expression of several M1 and M2 markers in IL-10^*−/−*^ and WT macrophages in CIA. With respect to M1 markers, we examined the M1-related genes TNF-α, IL-1β, IL-6 and iNOS (Figure 6D) and found that all had significantly elevated expression in macrophages of IL-10^*−/−*^ mice compared to those of WT mice. However, M2-associated markers such as IL-1ra, CD206 and IL-13 (Figure 6D) were not altered in either WT or IL-10^*−/−*^ macrophages. These results suggest that IL-10 deficiency might promote macrophage polarization toward the M1 phenotype and increase the expression of M1-mediated proinflammatory cytokines, which amplify the RA inflammation response.

## Discussion

Previous researchers have clearly demonstrated that inflammatory cytokines are essential for the development of RA [1,2] and that taking control of their secretion and action can suppress immune response, delay the development of RA and reduce the consequences of RA. IL-10, an anti-inflammatory cytokine produced by both macrophages and T cells, has been shown to play an important role in the regulation of inflammatory cytokine expression and their function. To assess the functions of IL-10 in CIA, we used C57BL/6 IL-10^−/−^ mice and WT mice as animal models to induce arthritis. Compared to WT mice, IL-10^−/−^ mice with CIA were confirmed by histological features to have a higher degree of inflammation in synovial tissues and higher levels of IL-17, IFN-γ, IL-6 and IL-1β in serum (Figure 1). Recent findings revealed that IL-10 signaling in T cells dampened the pathogenesis of CIA by inhibiting the expression of IL-17 [8,9]. The function of IL-10 in macrophages in CIA has not yet been studied, however. The results of our present experiments show that IL-10 inhibited the expression of IL-17 and its transcription factor RORγt in F4/80^+^ macrophages *in vitro* and *in vivo*. Similarly, IL-17 and RORγt expression was enhanced in IL-10^−/−^ mice and IL-10 receptor–deficient (IL-10R^−/−^) CD11b^+^ macrophages *in vitro*[25]. More importantly, IL-10^−/−^ mice had a significantly higher number of IL-17-producing F4/80^+^ macrophages in the synovium in CIA than WT mice did. Thus, our present findings indicate previously unrecognized IL-10 signaling in F4/80^+^ macrophages, which suppresses IL-17 and RORγt expression in F4/80^+^ macrophages. Given the substantial IL-17 expression in F4/80^+^ macrophages in the synovial tissues in CIA, IL-10 restriction of the IL-17 inflammation response in these cells might alleviate autoimmune inflammation during RA development.

Previously published research results suggest that several cell types, including γδT cells and mast cells [8,37], serve as sources of IL-17 in RA. Suurmond *et al*. showed that macrophages express IL-17 in RA and osteoarthritis synovium [37]. The critical effect of macrophages in RA should not be ignored, because the abundance and activation of macrophages in the inflaming synovial membrane/pannus significantly correlate with the severity of RA [14]. Although CD11b^+^ is a marker in murine macrophages, it is also highly expressed on the surface of many leukocytes, including monocytes, neutrophils, natural killer cells and granulocytes [38]. F4/80, a glycoprotein, has been established as a specific cell surface marker for murine macrophages [39,40]. The expression of F4/80 cells in various inflammatory tissues and disease models has been studied extensively [41]. F4/80 macrophages have been found to express higher levels of the Th1 cytokine IL-12 in inflammatory skin tissues than in healthy skin tissues [42]. F4/80 macrophages in the intestinal mucosa in Crohn’s disease patients were found to be much higher than in healthy individuals [43]. Our present results show that F4/80 macrophages had much higher IL-17 expression in the cell cultures stimulated by LPS than in the unstimulated cell cultures. Recently, it has been reported that the expression of IL-17 was significantly increased in CD11b^+^ cells of IL-10^−/−^ and IL-10R^−/−^ mice compared to WT mice [25]. However, how IL-10 regulates IL-17 expression in macrophages, especially in specific groups of macrophage (F4/80 macrophages), remains to be clarified. In addition, how this regulation affects the pathogenesis of CIA has not yet been studied. Our results show that the levels of IL-17 in the F4/80 macrophages of the peritoneum and joint tissues in IL-10^−/−^ mice were significantly higher than those in WT mice (Figures 3C, Figure 4A and 4B), which implies that IL-10 suppresses IL-17 expression in F4/80 macrophages in CIA. Moreover, immunofluorescence microscopy confirmed that the number of IL-17^+^ F4/80 macrophages was substantially increased in the synovial tissue of IL-10^−/−^ mice with CIA (Figure 4D), which suggests that IL-10 might play a role in local regulation of the production of IL-17 in macrophages in CIA. The number of IL-17^+^ F4/80 macrophages in synovial tissues increased in tandem with the severity of synovial hyperplasia, cartilage damage and bone erosion. These data imply that the markedly increased number of IL-17^+^ F4/80 macrophages in local joint tissue accelerated the pathogenesis of RA.

It is worth noting that IL-17 could induce the production of other proinflammatory cytokines, such as IL-6 and IL-1β, which cooperate to orchestrate inflammation of RA development [44]. Importantly, in our experiments, IL-10^−/−^ mice with CIA had significantly higher expression of IL-6 and IL-1β than WT mice with CIA did (data not shown). The results of our experiments indicate that IL-10 deficiency drove IL-17 production in macrophages in CIA joints, we cannot ignore the increased production of IL-17 in other cell types in synovial tissues of IL-10^−/−^ mice. For instance, it was recently reported that IL-10 signaling in T cells is critical for dampening the pathogenesis of CIA by recruitment of IL-17^+^ γδT cells [8]. Furthermore, the majority of IL-17 expression seems to occur in mast cells of the synovium in RA [37].

An important question arose from our data: How is the IL-10 signaling pathway involved in the mediation of IL-17 expression in macrophages of the synovium in CIA? RORγt, a specific transcription factor essential for the development of Th17 cells, induces transcription of the genes encoding IL-17 [45,46]. Mice with RORγt-deficient T cells are resistant to the induction of autoimmune disease and lack tissue-infiltrating Th17 cells [46]. Interestingly, recently reported data demonstrate that RORγt expression is enhanced in the spleen cells and thioglycollate-elicited cells of IL-10^−/−^ mice *in vitro*[25]. However, whether IL-10 is involved in the regulation of RORγt expression in joint F4/80^+^ macrophages remains to be studied. In agreement with our hypothesis, the results of our experiments show that IL-10^−/−^ F4/80^+^ macrophages significantly increased the expression of RORγt compared to WT mice F4/80^+^ macrophages *in vitro* and *in vivo* (Figures 3E, 3F, 5A and 5C). These data demonstrate that inhibited IL-17 production by IL-10 in macrophages ois associated with suppression of RORγt expression.

At this stage, we still cannot completely elucidate the mechanisms underlying the upregulation of IL-17 expression in macrophages of CIA IL-10^−/−^ mice. First, the exact IL-10 signaling cascade involved in the downregulation of IL-17 expression has not yet been fully illuminated. Second, previously published studies have shown that IL-10 exerts its anti-inflammatory effects in macrophages by activating signal transducer and activator of transcription 3 (STAT3) and STAT1 [7,47,48], but the puzzle surrounding relationships between STAT3, STAT1 and RORγt remains to be resolved. Further investigation is necessary to completely understand the mechanism of IL-10 signaling in the mediation of IL-17 expression in macrophages in CIA. Collectively, to the best of our knowledge, we elucidate for the first time that IL-10 attenuation of the development of CIA is associated with suppression of IL-17 and RORγt production in macrophages.

The initiation, development and resolution of RA are determined by a dynamic balance between effector and regulatory immune cell populations. Activated macrophages contribute to RA pathogenesis by secreting proinflammatory cytokines and thereby take part in the Th17/Th1 response [49,50]. The polarization of macrophages into the M1 or M2 phenotype depends on the cytokine milieu in the tissue. Macrophages develop into the proinflammatory M1 phenotype in response to IFN-γ and LPS, whereas the anti-inflammatory M2 phenotype is induced by Th2 cytokines, including IL-4 and IL-13. Of note, IL-10, an anti-inflammatory Th2 cytokine, can drive macrophage polarization toward an anti-inflammatory M2 phenotype and can block the differentiation of proinflammatory M1 macrophages [19,35]. An important role of the M1 subset of macrophages in exacerbating RA was demonstrated previously [49,50]. Our data further confirm that IL-10 deficiency drives macrophage polarization toward the proinflammatory M1 phenotype and increases the expression of M1-mediated proinflammatory cytokines, which contributes to the RA inflammatory response. However, macrophages are plastic cells, because they can switch from an activated M1 state back to M2, and vice versa, under different conditions. Further studies are needed to examine the change of macrophages phenotype in the different stages of RA disease and the molecular mechanism of IL-10-mediated attenuation of CIA by repressing polarization of the proinflammatory M1 phenotype.

## Conclusion

The results of our present study provide evidence for the involvement of IL-10 in ameliorating the pathological process of CIA that is associated with inhibition of IL-17 and RORγt production in macrophages and repression of M1 macrophages. Without IL-10 signaling, macrophages, especially F4/80^+^ macrophages, lost their suppressive function, which in turn failed to control IL-17-triggering inflammatory responses in RA, especially locally in synovial tissues. Enhancement of IL-10 signaling in macrophages might serve as a therapeutically sound approach to the treatment of RA as well as other autoimmune inflammatory diseases.

## Abbreviations

CCR7: C-C chemokine receptor type 7; CD163: Cluster of differentiation 163; CD206: Mannose receptor; CIA: Collagen-induced arthritis; CII: Chicken type II collagen; CXCL: Chemokine (C-X-C motif) ligand; DAPI: 4′,6-diamidino-2-phenylindole; DC: Dendritic cell; ELISA: Enzyme-linked immunosorbent assay; FBS: Fetal bovine serum; FITC: Fluorescein isothiocyanate; IFN-γ: Interferon γ; IgG: Immunoglobulin G; IL: Interleukin; IL-10−/−: Interleukin 10–knockout; IL-1ra: IL-1 receptor antagonist; iNOS: Inducible nitric oxide synthase; LPS: Lipopolysaccharide; M1: Classically activated macrophage; M2: Alternatively activated macrophage; PBS: Phosphate-buffered saline; PE: Phycoerythrin; RA: Rheumatoid arthritis; RORγt: Retinoid-related orphan receptor γt; STAT: Signal transducer and activator of transcription; TH: T helper cell; TNF-α: Tumor necrosis factor α; WT: Wild type.

## Competing interests

The authors declare that they have no competing interests.

## Authors’ contributions

LY participated in the conception and design of the study; data acquisition, collection, analysis and interpretation; manuscript writing; critical revision of the manuscript. ZW participated in macrophage isolation, CIA induction experiments, Western blot analysis and critical revision of the manuscript. YL, BC and TY participated in macrophage isolation, RNA and cDNA experiments, RT-PCR analysis and manuscript writing. LLiu participated in immunofluorescence histochemistry, confocal and immunofluorescence microscopy and manuscript writing. JZ, YM and SX participated in flow cytometric analysis, collection of serum and synovial fluid samples and critical revision of the manuscript. LD and LLi participated in ELISA experiments, statistical analysis, manuscript drafting and critical reading of the manuscript. ZH participated in the conception and design of the study, data and statistical analysis, manuscript writing, critical revision of the manuscript and correspondence with the editor of the journal. All authors read and approved the final manuscript.
